# A Balance Equation Determines a Switch in Neuronal Excitability

**DOI:** 10.1371/journal.pcbi.1003040

**Published:** 2013-05-23

**Authors:** Alessio Franci, Guillaume Drion, Vincent Seutin, Rodolphe Sepulchre

**Affiliations:** 1 INRIA Lille-Nord Europe, Orchestron Project, Villeneuve d'Ascq, France; 2 Department of Electrical Engineering and Computer Science and GIGA Research, University of Liège, Liège, Belgium; 3 Neurophysiology Unit, GIGA Neurosciences, University of Liège, Liège, Belgium; University of Oxford, United Kingdom

## Abstract

We use the qualitative insight of a planar neuronal phase portrait to detect an excitability switch in arbitrary conductance-based models from a simple mathematical condition. The condition expresses a balance between ion channels that provide a negative feedback at resting potential (restorative channels) and those that provide a positive feedback at resting potential (regenerative channels). Geometrically, the condition imposes a transcritical bifurcation that rules the switch of excitability through the variation of a single physiological parameter. Our analysis of six different published conductance based models always finds the transcritical bifurcation and the associated switch in excitability, which suggests that the mathematical predictions have a physiological relevance and that a same regulatory mechanism is potentially involved in the excitability and signaling of many neurons.

## Author Summary

Understanding the changing electrophysiological signatures of neurons in different physiological and pharmacological conditions is a central focus of experimental electrophysiology because a key component of cell signaling in the nervous system. Computational modeling may assist experimentalists in this quest by identifying core mechanisms and suggesting pharmacological targets from a mathematical analysis of the model. But a successful interplay between experiments and mathematical predictions requires new analysis tools adapted to the complexity of high-dimensional computational models nowadays available. We use bifurcation theory to propose a mathematical condition that can detect an important switch of neuronal excitability in arbitrary conductance-based neuronal models and we illustrate its physiological relevance in six published state-of-the art models of different neurons.

## Introduction

Detailed computational conductance-based models have long demonstrated their ability to faithfully reproduce the variety of electrophysiological signatures that can be recorded from a single neuron in varying physiological or pharmacological conditions. But the predictive value of a computational model is limited unless its analysis sheds light on the core mechanisms at play behind a computer simulation. Because conductance-based models are nonlinear dynamical models, their analysis often requires a drastic reduction of dimension. The reduced model is amenable to the geometric methods of dynamical systems theory, but the mathematical insight is often gained at the expense of physiological interpretability; hence the need for methodological tools that can relate mathematical predictions of low-dimensional models to physiological predictions in detailed conductance based models.

In recent work [1], we used phase plane analysis and dynamical bifurcation theory to characterize in *reduced-order* neurodynamics models a switch of excitability that is consistent with many physiological observations. More precisely, a transcritical bifurcation governed by a single parameter was shown to organize a switch from restorative excitability, extensively studied in most models inspired from the Hodgkin-Huxley model, to regenerative excitability whose distinct electrophysiological signature include spike latency, plateau oscillations, and afterdepolarizeation potentials.

The main contribution of the present paper is to show that this transcritical bifurcation, and the associated excitability switch, exist in a number of *high-dimensional* conductance-based models and that the resulting mathematical predictions have physiological relevance. Although purely mathematical in nature, the detection of the transcritical bifurcation relies on an ansatz that leads to a simple physiological interpretation: the switch of excitability is determined by a balance between restorative (those providing a negative feedback) and regenerative (those providing a positive feedback) ion channels at the resting potential. Because this simple balance equation can take many different physiological forms, it is potentially shared by very different neurons.

We use the balance equation to provide an algorithm to trace the transcritical bifurcation in arbitrary conductance-based models. We apply the algorithm to detailed conductance-based models of six neurons known to exhibit drastic changes in their electrophysiological signatures depending on environmental conditions: the squid giant axon [2], the dopaminergic neuron [3], the thalamic relay neuron [4], the thalamic reticular neuron [5], the aplysia R15 model [6], and the cerebellar granule cell [7]. In each case, the algorithm identifies a transcritical bifurcation that occurs close to the nominal model parameters and its predictions are consistent with experimental observations.

After defining a novel classification of ion channels based on their restorative or regenerative nature, we briefly review the planar model presented in [1] and how its transcritical bifurcation qualitatively captures the switch between restorative and regenerative excitability. As a generalization of this low-dimensional case, we mathematically construct the same bifurcation in generic conductance based models and derive the balance condition determining the regenerative or restorative nature of the model. This construction and its electrophysiological predictions are firstly illustrated on the squid giant axon. An algorithm for generic conductance-based models is subsequently derived and different models analysed.

## Results

### Slow restorative and slow regenerative ion channels

Conductance-based models of neurons describe the dynamic interaction between the membrane potential 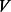 and - possibly many - gating variables that control the ionic flow through the membrane. The gating of ion channels occurs on many different timescales. However, gating timescales can be grouped in three families, according to their influence on neuronal excitablity [8]:

- **Fast gating variables:** These variables have a time-constant in the millisecond range. They generate the rapid regenerative upstroke of an action potential. Prominent representatives of this family are the activation gating variables of fast voltage-gated sodium channels (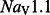 to 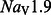).
- **Slow gating variables:** These variables have a time constant 5 to 10 times larger than fast gating variables. They influence the spike initiation, downstroke, and the afterspike period. They are key players of neuronal excitability. Prominent representatives of this family are the activation gating variables of delayed rectifier potassium channels (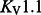 to 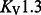, 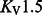 to 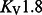, 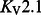, 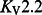, 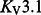, 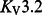, 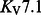 to 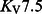, 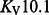) and the activation gating variables of all calcium channels (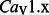, 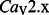, 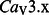).
- **Ultra-Slow (adaptation) variables:** These variables gate too slowly to be strongly activated by single action potentials. They modulate neuronal excitability only over periods of many action potentials. Prominent representative of this family are the inactivation gating variables of transient calcium channels (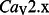, 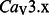). Ultra-slow variables might also include non gating variables. For instance, the intracellular calcium concentration 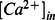, which modulates the conductance of calcium-regulated channels.

In view of their importance for neuronal excitability, we focus only on slow gating variables to classify ion channels: when the slow channel provides a negative feedback on membrane potential variations, we term the associated channel a slow restorative ion channel. When the slow variable instead enhances a potential variation by positive feedback, the associated ion channel is termed slow regenerative (a characterization in terms of partial derivatives is postponed to the next sections). Ion channels that do not possess a slow gating variable are neither restorative nor regenerative and are called neutral. Neutral ion channels solely regulate the “quantity” of excitability without affecting its “quality”.

Table 1 shows a classification of many known ion channels according to this criterion. Not surprisingly, potassium channels are the main representatives of slow restorative ion channels. By increasing the total outward current, their activation induces a negative feedback on membrane potentials variations that is responsible for neuron repolarization. On the other hand, physiologically described calcium channels are all slow regenerative. Their activation induces an increase of the total post-spike inward current, in contrast to potassium channels. This is the source, for instance, of afterdepolarization potentials (ADP). Interestingly, sodium channels can be either restorative, regenerative, or neutral according to their fast transient, resurgent, or persistent behavior, respectively.

**Table 1:** Classification of ion channels according to their gating kinetics.

Ion Channel	Gating Kinetics	Classification
**Sodium Channels**		
Transient	FA, SI - Negative feedback via SI	Slow restorative
Persistent	FA - No slow gating variable	Neutral
Resurgent	SA, USI - Positive feedback via SA	Slow regenerative
**Calcium Channels**		
L-type	SA - Positive feedback via SA	Slow regenerative
T-type	SA, USI - Positive feedback via SA	Slow regenerative
N, P/Q, R-type	SA, USI - Positive feedback via SA	Slow regenerative
**Potassium Channels**		
Delayed Rectifiers	SA - Negative feedback via SA	Slow restorative
KCNQ	USA - No slow gating variable	Neutral
eag/erg	USA - No slow gating variable	Neutral
A-type	FA, SI - Positive feedback via SI	Slow regenerative
BK	SA - Negative feedback via SA	Slow restorative
HCN	SA - Negative feedback via SA	Slow restorative

Activation and inactivation variables are distributed in three groups: fast, slow, and ultra-slow (adaptation). Slow variables are defined as restorative (resp. regenerative) if they induce a negative (resp. positive) feedback on membrane potential variations. An ion channel that posses a slow restorative variable is called “slow restorative channel”, and similarly for slow regenerative channels. Channels that do not posses a slow variable are called “neutral channels”. This classification might change for a given channel for some channel subtypes.

FA: fast activation, FI: fast inactivation, SA: slow activation, SI: slow inactivation, USA: utraslow activation, USI: ultraslow inactivation.

It is important to observe that the restorative (resp. regenerative) nature of channels is not solely linked to the outward (resp. inward) nature of the current. For instance, transient sodium channels (although responsible for the regenerative spike upstroke) are slow restorative, because their slow variable inactivates an inward current, inducing a negative-feedback on membrane potential variations. Similarly, potassium channels can be slow regenerative when their slow inactivation massively decreases the outward current, like in the case of A-type potassium channels.

Although elementary, the classification above seems novel. It is motivated by the central message of this paper, that the balance between regenerative and restorative ion channels in slow timescale determines its neuronal excitability type. In the remainder of the paper, we simply write restorative (resp. regenerative) for slow restorative (resp. slow regenerative) channels.

### Restorative and regenerative excitability in planar models

Planar models - that only consist of two state variables - have been instrumental to study excitability since the early days of neurodynamics [9], [10]. Empirical planar reductions of conductance based models only retain the (fast) voltage variable 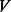 and one slow gating variable 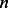. Fast gating variables are set to steady-state (i.e. their fast variation is approximated as instantaneous), adaptation variables are treated as slowly varying parameters, and the sole gating variable 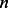 aggregates all slow variables, expressing each of them as a (curve-fitted) static function of 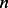.

**Figure 1:**
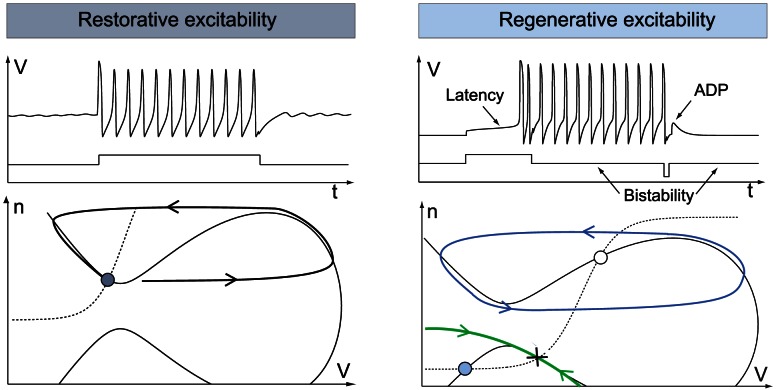
The bottom figures illustrate the typical phase portrait of restorative (left) and regenerative (right) excitability. The dark (resp. light) blue circle denotes a stable restorative (resp. regenerative) steady state 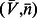. The thin full (resp. dashed) curve is the voltage (resp. slow variable) nullcline. The saddle point in the right phase portrait is represented by a cross and its separatrix as the green oriented curve. The stable limit cycle surrounding the unstable fixed point (represented as a circle) is represented by the blue oriented curve. The thick curve in the left phase portrait represents the typical trajectory associated to the generation of an action potential. The top figures illustrate the typical accompanying electrophysiological responses to step variations of current.

Motivated by the phase portrait of such an empirical reduction of the Hodgkin-Huxley model augmented with a calcium channel [11], our recent study [1] explores the neuronal excitability of the planar model
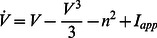
(1a)

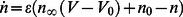
(1b)whose phase portraits are reproduced in Fig. 1 for two distinct values of the parameter 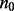 (an indirect representation of the calcium conductance in the high-dimensional model). The parameter 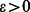 characterizes the time-scale separation between 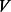 and 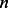. The function 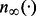 has the standard sigmoid shape of conductance-based models and 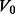 is the half-activation potential. The typical step responses of (1) are also reproduced in Fig. 1.

The spike generation mechanism in the phase portrait of Fig. 1 left is reminiscent of the of FitzHugh-Nagumo model and of the physiologically grounded planar reduction of Hodgkin-Huxley model by Rinzel [10]. It is associated to a reversible and sudden switch from resting to spiking and has been studied extensively, with finer distinctions depending on the mathematical nature of the underlying bifurcation [12]. For further reading, see [13], [14, Section 7.1.3], and references therein.

The phase portrait in Fig. 1 right is in sharp contrast in that the electrophysiological response to a current input exhibits spike latency, plateau oscillations, and after depolarization potential (ADP). This specific signature, experimentally observed in many families of neurons, is fundamentally associated to the bistability illustrated in the phase portrait: namely, the robust coexistence of two stable attractors (a hyperpolarized resting potential and a limit cycle of periodic action potentials) and a saddle-separatrix that sharply separates their basins of attraction. The time evolution shown in the top figure is a consequence of this phase portrait and cannot be observed in FitzHugh-Nagumo like phase portraits. The distinction between the two phase portraits, the associated excitability types, and their relation with Hodgkin's excitability classification [15] are further discussed in [1] and later in the paper.

**Figure 2:**
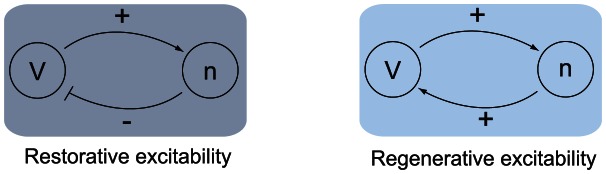
Block diagram illustration of restorative and regenerative excitability in planar models.

A simple mathematical distinction between the two phase portraits shown in Fig. 1 is drawn from the Jacobian linearization of the model at the stable resting point 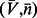:
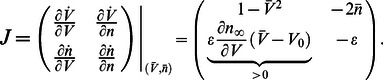
The product of the partial derivatives 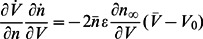 is negative on the left phase portrait (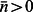), capturing the restorative nature of the gating variable, whereas it is positive on the right phase portrait (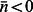), capturing the regenerative nature of the gating variable. This difference is schematized in the block diagrams of Fig. 2. Accordingly, excitability in planar models is called restorative (resp. regenerative) when the gating variable provides negative (resp. positive) feedback close to the resting point:

#### Planar restorative excitability

The model is said to be restorative at steady state if:
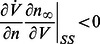



#### Planar regenerative excitability

The model is said to be regenerative at steady state if:
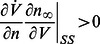



The planar model (1) can smoothly switch from restorative to regenerative excitability, with a transition occurring for 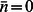, or, in algebraic terms,
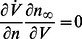
(2)


A convenient way to algorithmically track this excitability switch is to use bifurcation analysis and to impose that the critical condition (2) coincides with a bifurcation of the model, which imposes the additional algebraic condition
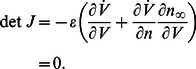
(3)


**Figure 3:**
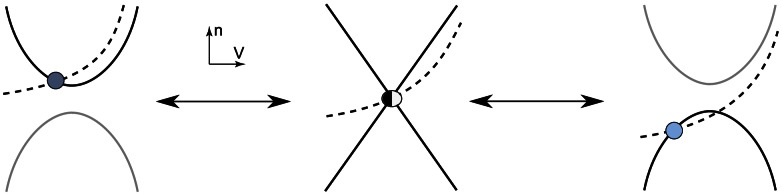
A continuous deformation from the restorative phase portrait of Fig. 1 left to the regenerative phase portrait of Fig. 1 right involving a transcritical bifurcation [17, Section 3.2] determined by the algebraic conditions (2) and (3). The dark blue circle represents a restorative stable steady-state, the light blue circle a regenerative stable steady-state, and the half-filled circle represents the transcritical bifurcation which separates the restorative and regenerative regimes.

Simultaneously imposing (2) and (3) implies
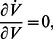
(4)which, in geometrical terms, corresponds to the transcritical bifurcation obtained for 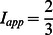 and illustrated in Fig. 3.

**Figure 4:**
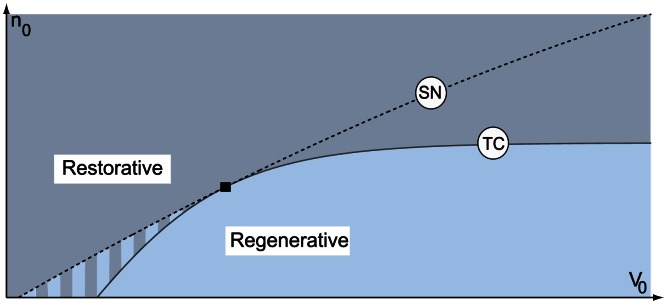
Excitability types in model (1). SN denotes the saddle-node bifurcation, TC the transcritical bifurcation. The black square denotes the pitchfork bifurcation organizing center. Varying 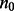 and 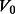 the model switches between excitability types.

The theory of bifurcation unfolding is further exploited in [1] in order to classify all excitability types associated to the planar model in Fig. 1. This analysis results in five different types of excitability obtained by varying the two parameters 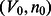 around the singular phase portrait of Fig. 3, center. The parameter 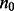 acts in particular as the sole regulator of the balance between regenerative and restorative excitability by shifting the 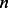-nullcline up and down: a positive 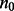 corresponds to a phase portrait as in Fig. 3 left, whereas the phase portrait of Fig. 3 right is obtained for sufficiently negative 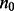. An early graphical manifestation of such phase portraits in conductance-based models is in [16, Figs. 17,18, and 19]. Figure 4 delineates the two types of excitability in a two parameter chart. It contains the two types of excitability discussed above. The transition from restorative to regenerative excitability is always through a transcritical bifurcation. In addition, some paths traverse a small mixed region where a down regenerative steady state and an up restorative steady state coexist.

Our main contribution in the present paper is to show that the diagram in Fig. 4 is not an artifact of planar reduction but captures excitability transitions that can be algorithmically tracked in conductance-based models of arbitrary dimension by imposing a simple physiologically relevant algebraic condition.

### Restorative and regenerative excitability in conductance based models

We start by grouping gating variables of a given conductance-based model according to their time scales. The family 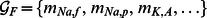 collects fast gating variables. The gating variable 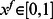 denotes an arbitrary member of this family. Similarly, the family 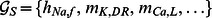 collects slow gating variables 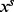, whereas 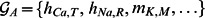 collects adaptation variables 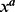. For a given ion channel type 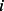, the standard notation 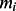 (resp. 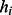) is adopted for the activation (resp. inactivation) gating variable of the associated ionic current 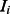. With these notations, a general neuron conductance-based model reads
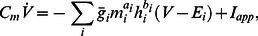
(5a)

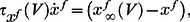
(5b)

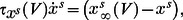
(5c)

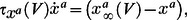
(5d)where the sum in (5a) is over all ion channels in the model, and (5b),(5c),(5d) hold for all the associated fast, slow, and adaptation variables, respectively. The activation (resp. inactivation) functions 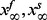 are strictly monotone increasing (resp. decreasing) sigmoids. In the forthcoming analysis, all adaptation variables are treated as constant parameters, that is, their slow evolution is neglected.

We will detect a switch from restorative to regenerative excitability by mimicking the two-dimensional algorithm of the previous section. We first impose the bifurcation condition 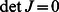, where 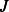 denotes the Jacobian of the subsystem (5a),(5b),(5c). The algebraic condition writes
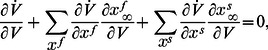
(6)where the sums are over all fast and slow variables, respectively. The particular form of equation (6) is a direct consequence of the specific structure of conductance-based models, that is, parallel interconnection of two-dimensional feedback loops involving the voltage dynamics (6a) and one of the gating variable dynamics (6b),(6c).

As for the planar model (1), we track the switch between restorative and regenerative excitability by imposing the high-dimensional equivalent of the balance condition (2). We therefore look for solutions of (6) satisfying the ansatz
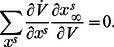
(7)


The two conditions (6) and (7) now imply
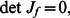
(8)where 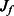 denotes the Jacobian of the fast subsystem (5a),(5b). We show in Supplementary Material S1 that the corresponding bifurcation is necessarily transcritical [17, Section 3.2].

The singularity condition (8) is the high-dimensional counterpart of the 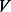-nullcline self-intersection in the planar model. It reflects the geometric nature of the transcritical bifurcation, that is, a robust geometrical object that exists independently of the timescale separation and persists in the singular limit of an infinite timescale separation, regardless of the system dimension. Our ansatz makes the proposed analysis robust against the model time constants. The time constants are only used to classify the gating variables in the three physiological groups.

We split 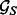 in two subfamilies: 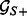, which contains *regenerative* slow gating variables 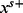, and 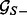, which contains *restorative* slow gating variables 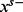. The balance condition (7) is then rewritten as
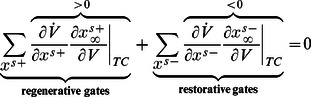
(9)to express a balance between restorative and regenerative ion channels. It is the high-dimensional counterpart of (2) and it provides a rigorous high-dimensional generalization of restorative and regenerative excitability:

#### Restorative excitability

The model is said to be restorative at steady state if:
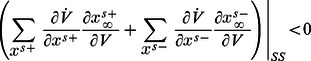



#### Regenerative excitability

**Figure 5:**
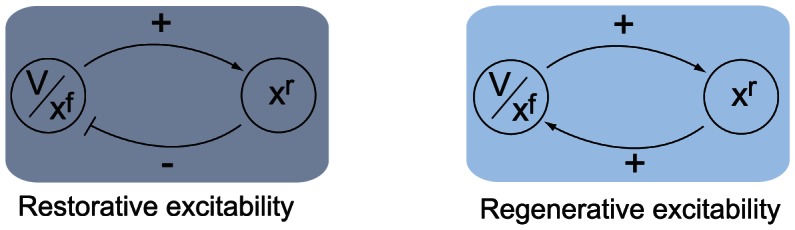
Block diagram illustration of restorative and regenerative excitability in conductance based models.

The model is said to be regenerative at steady state if:
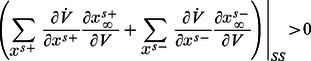
A block diagram representation is illustrated in Fig. 5. The insight provided by the planar model of the previous section predicts that the switch of excitability detected by the balance equation (9) will lead to the accompanying distinct electrophysiological signatures of Fig. 1.

### Tracking excitability switches in the squid giant axon

The Hodgkin-Huxley (HH) model [2] provides a non-physiological, but historical and experimentally verified tutorial for tracking a switch of excitability in conductance based models. The model reads
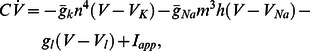
(10a)

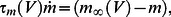
(10b)

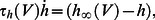
(10c)

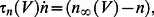
(10d)where 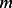 is the fast sodium channel activation while the sodium channel inactivation 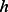 and the potassium channel activation 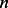 are the slow gating variables. We set all time constants to one, because this simplification has no effects on the algebraic conditions (7) and (8). The Jacobian of (10) reads
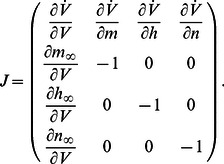
(11)The upper-left block is the Jacobian of the fast 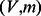 subsystem. Imposing the singularity condition (8) yields
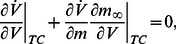
(12)while the balance equation (9) reads
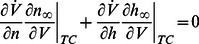
(13)Note that (12) and (13) imply the bifurcation condition 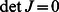 in (11).

At first sight, the balance condition (13) cannot be satisfied because both sodium and potassium channels are restorative channels according to their corresponding kinetics in the model, and in agreement with our proposed classification. This is consistent with the fact that the excitability of the HH model is always restorative in physiological conditions.

However, it was long recognized [18] that potassium channels can generate an inward current at steady-state if the extracellular 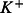 concentration is sufficiently large. Indeed, any change in extracellular potassium concentration induces a change in the potassium reversal potential, as expressed by the Nernst equation. This suggests to use the potassium reversal potential 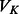 as a bifurcation parameter in HH model in order to satisfy the balance equation
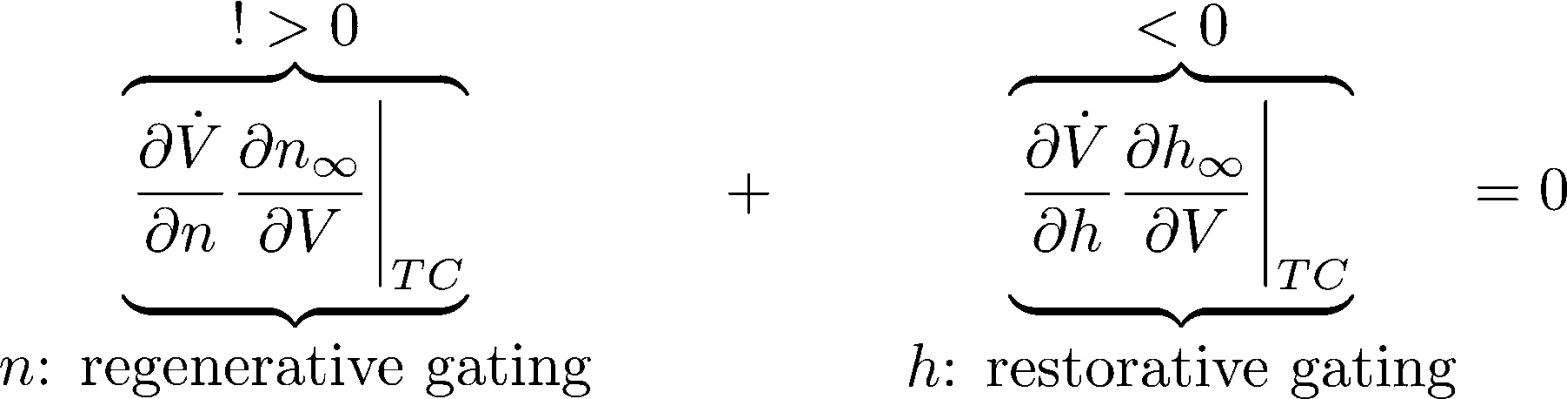
(14)where potassium now acts as a regenerative gating variable provided that 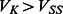. Physiologically, condition (14) imposes that the potassium Nernst potential is large enough for the regenerativity of the potassium activation to balance the restorative effects of the sodium current inactivation.

**Figure 6:**
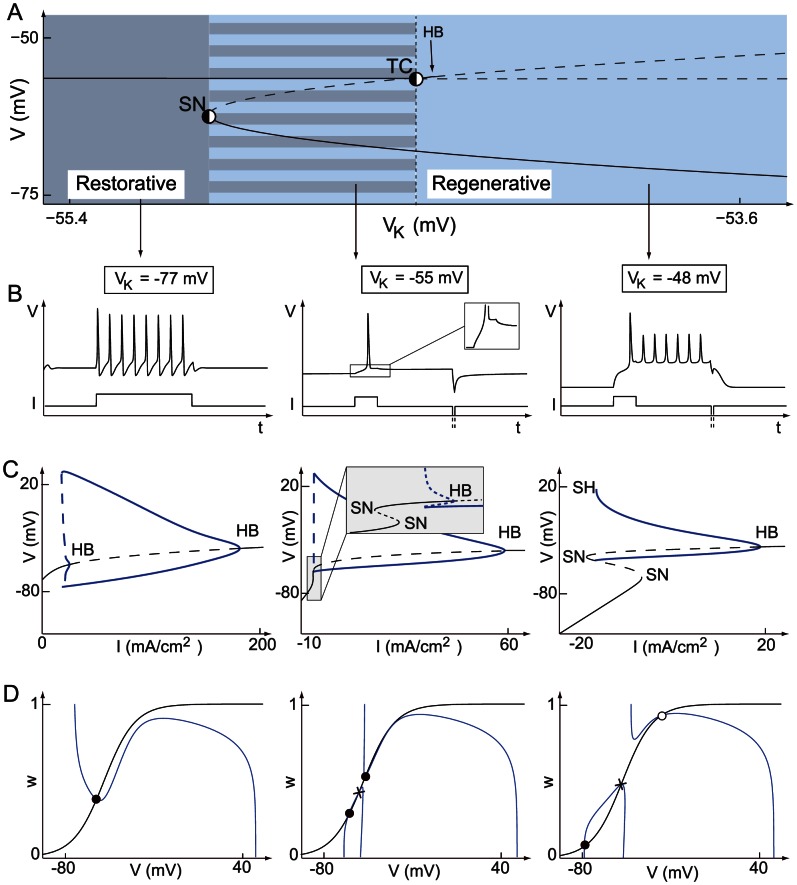
Variations of the potassium reversal potential 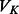 induce excitability switches in the Hodgkin-Huxley model. **A.** Bifurcation diagram of the HH model with 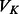 as the bifurcation parameter. TC denotes a transcritical bifurcation, SN a saddle-node bifurcation, HB a Hopf bifurcation. Branches of stable fixed points are represented as solid curves, whereas branches of saddle points and unstable points as dashed curves. **B.** Electrophysiological responses of the model for three different values of 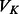, corresponding to three different excitability types (restorative, mixed, and regenerative, from left to right). **C.** Bifurcation diagrams with the applied current as the bifurcation parameter for the same three values of 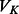 as in **B**. Black (resp. blue) full curves represent branches of stable steady-states (resp. limit cycles), black dashed curves branches of saddle and unstable steady-states. Branches of unstable limit cycle are drawn as dashed blue curves. HB denotes a Hopf bifurcation, SN a saddle-node bifurcation, and SH a saddle-homoclinic bifurcation. **D.** Phase portraits of reduced HH model proposed by Rinzel in [10] for the same three values of 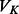 as in **B,C**. Blue full curves denote the 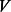-nullclines and black full curves the 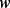-nullclines, where 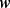 denotes the slow variable of the reduced model. Filled circles denote stable steady-states, crosses saddle points, and circles unstable steady-states.

The two conditions (12) and (14) can numerically be solved to determine the critical values 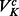 and 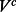. The value of the applied current at the transcritical bifurcation is then determined from (10a), which gives
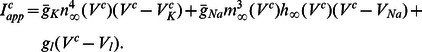
The numerical bifurcation diagram in Fig. 6A confirms the transcritical bifurcation at 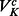. That bifurcation diagram is drawn by varying 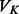 together with applied current 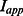, following the affine reparametrization described in Supplementary Material S1. More precisely,
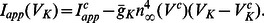
Mathematically, this reparametrization imposes one of the defining conditions of the transcritical bifurcation. Physiologically, its effect is to keep the net current constant at steady-state 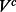 (*i.e.* 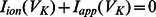): as 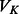 is varied, the observed switch in the excitability type does not rely on changes in the net current across the membrane, but solely on changes in its dynamical properties.

The bifurcation diagram in Fig. 6A provides informations on the model excitability also far from the transcritical values. For highly hyperpolarized 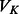, the model is purely restorative and exhibits the typical excitable behavior of the original Hodgkin-Huxley model [1, Figure 8]. As 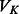 is increased, a stable regenerative steady-state is born in a saddle-node bifurcation. At this transition, the system switches to a mixed excitability type. Short current pulses let the system switch between the depolarized restorative stable steady state and the hyperpolarized regenerative stable steady state (Fig. 6B,middle). The associated bifurcation diagram and phase portrait are reproduced in Fig. 6C,D,middle. Finally, a further increase of 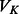 lets the restorative steady state exchange its stability with a (regenerative) saddle at the transcritical bifurcation (and, soon after, lose stability in a Hopf bifurcation) and the system switches to regenerative excitability. The regenerative steady state coexists in this case with the spiking limit cycle attractor. Current pulses switch the system asymptotic convergence between the two attractors (Fig. 6B,right). The associated bifurcation diagram and phase portrait are reproduced in Fig. 6C,D,right.

The same qualitative excitability switch was described by Rinzel in [10], who linked the appearance of a bistable behavior to the inward nature of potassium current at steady-state for sufficiently depolarized 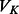. *In vitro* recordings of the squid giant axon with isotonic extracellular 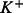 concentration show the same transition [18].

### Tracking excitability switches in conductance-based models

The mathematical analysis of the previous section follows an algorithm that allows to detect a transcritical bifurcation in generic conductance based models of arbitrary dimension and to track associated excitability switches. The steps of the algorithm are summarized in Table 2. For simplicity and conciseness, we restrict our attention to the modulation of only one regenerative ionic current. However, a similar algorithm can be written for an arbitrary modulation of ionic currents (by variation of maximal conductance(s), adaptation variable(s), or reverse potential(s)) that brings the model to the balance expressed in (9).

**Table 2:** Algorithm for the detection of a transcritical bifurcation in generic conductance-based models via modulation of a regenerative ionic current and computation of the excitability switch bifurcation diagram.

(i) **Classification of gating variables as fast (** 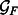 **), slow (** 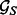 **), and adaptation (** 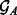 **) variables**
(i-a) Following Tab. 1, group gating variables in the three groups 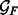 , 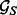 , and 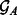 .
(i-b) Split 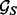 in regenerative 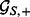 and restorative 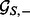 slow gating variables.
(i-c) If adaptation variables are present, set them to constant physiologically relevant values
(ii) **Balance equation and choice of the bifurcation parameter**
(ii-a) Select a regenerative ionic current 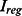 and the associated regenerative slow gating variable 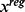 .
(ii-b) Write the balance equation
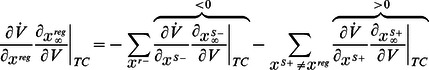 (b.eq.)
(ii-c) If 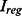 has an adaptation variable 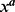 , pick it as the bifurcation parameter 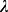 regulating the left hand side of (b.eq.), that is 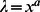 .
If 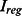 has no adaptation variable, pick 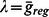
(iii) **Singularity condition and fixed point equation**
(iii-a) Solve (b.eq.) together with the singularity condition (8), in 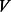 and 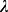 .
For numerical implementation, recall that the left hand side of (8) is proportional to
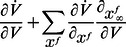
(iii-b) Plug the computed values 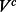 and 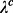 into the fixed point equation 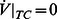 to compute the value of the applied current at the transcritical bifurcation ( 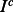 ).
(iv) **Tracking of excitability switches**
Change the applied current according to the equation
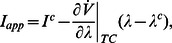
and compute the model bifurcation diagram with 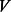 as the variable and 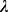 as the bifurcation parameter.

**Figure 7:**
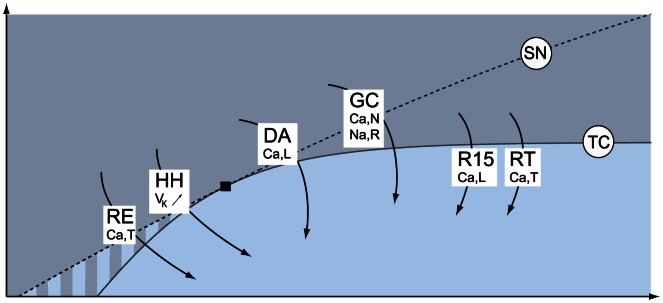
Modifications in the balance between restorative and regenerative channels induce excitability switches in conductance-based models. The figure sketches excitability switches of the Hodgkin-Huxley (HH) model [2], Aplysia's R15 neuron (R15) model [6], a dopaminergic (DA) neuron model [3], thalamic reticular (RT) and relay (RE) neuron models [4], [5], and a cerebral granule cell (GC) model [7] on the excitability parameter map computed for the two-dimensional model of [1]. All these conductance-based models can switch between restorative and regenerative excitability through the physiologically relevant regulation of specific ion channels.

For the sake of illustration, we apply this algorithm to a number of published conductance-based models and show that all these models can switch between restorative and regenerative excitability through a transcritical bifurcation, as sketched in Fig. 7. Figure 7 indicates two qualitatively distinct paths from restorative to regenerative excitability: one path traversing the mixed excitability region just described with Hodgkin-Huxley model (Fig. 6A) and one path switching directly from restorative to regenerative excitability through the TC bifurcation that will be illustrated on the dopaminergic neuron model.

#### Dopaminergic (DA) neuron model

Model equations and parameters are taken from [3]. This is the model that originally motivated [11].

- **Classification of gating variables as fast (** 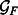 **), slow (** 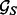 **), and adaptation (** 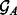 **) variables.** The model includes fast sodium channels (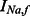), delayed-rectifier potassium channels (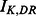), L-type calcium channels (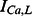), small conductance calcium-activated potassium (SK) channels (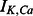), and calcium pumps (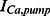). We classify model variables as follows

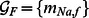

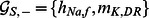
 and 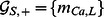

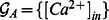

- In order to unfold excitability switches, SK channel density is set to zero, since SK channels drastically attenuate DA neuron excitability by activating a strong calcium regulated potassium current [19], [20], [21]. The intracellular calcium concentration is fixed at 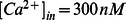.
- **Balance equation and choice of the bifurcation parameter.** The only source of regenerative excitability is provided by L-type calcium channels. The balance equation reads
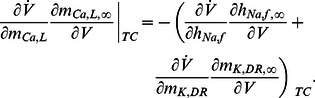
(15)

We use the L-type calcium channel density 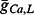 as the bifurcation parameter (*i.e.* 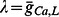).

Solving Steps (iii) and (iv) of the algorithm above gives the following results (in this and the subsequent tables, the reported critical values are not exact, but rounded to the last significant digit):
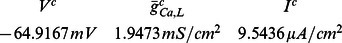
The critical value 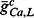 is roughly 1.5 times smaller than the nominal parameter value in [3], which is consistent with the observation that the original model exhibits regenerative excitability during SK channel blockade.

**Figure 8:**
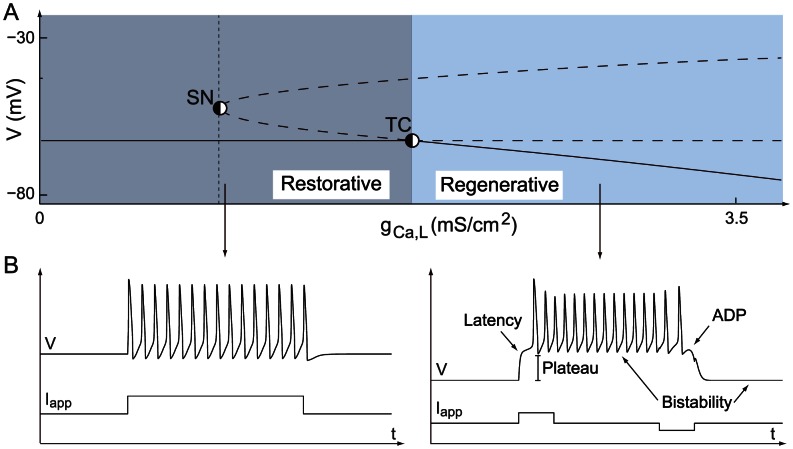
Variations of L-type calcium channel density 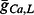 induce excitability switches in a model of DA neurons [**3**] **.** **A.** Bifurcation diagram of the model with 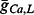 as the bifurcation parameter. TC denotes a transcritical bifurcation, SN a saddle-node bifurcation. Branches of stable fixed points are represented as solid curves, branches of saddle points and unstable points as dashed curves. **B.** Electrophysiological responses of the model to step inputs of excitatory/inhibitory current (the intracellular calcium concentration is fixed at 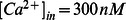, which is within the physiological range). For 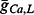 lower (resp. higher) than the critical value 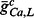, the model exhibits typical electrophysiological signature of restorative (resp. regenerative) excitability. The low 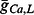 configuration corresponds to 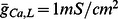, whereas the high 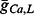 configuration corresponds to 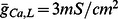.

The resulting bifurcation diagram is drawn in Fig. 8A. In addition to confirming the existence of a transcritical bifurcation for the computed values, it reveals the excitability switches induced by changes in L-type calcium channel density in this model: in the absence of L-type calcium channels, the model exhibits restorative excitability. As 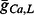 increases, a saddle point and an unstable node emerge at a saddle-node bifurcation, which induces no excitability switch. Further increase of 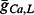 causes a transcritical bifurcation, where the stable point and the saddle exchange their stability. At this point, the stable steady-state becomes regenerative, and the model switches to regenerative excitability.

These excitability switches induce the predicted changes in the electrophysiological signatures, as illustrated in Fig. 8B. Whereas the DA neuron model instantaneously reacts to a step input of depolarizing current for 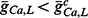, it exhibits spike latency, plateau oscillations and ADP as soon as 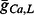 becomes higher than 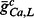. In addition, the model becomes strongly bistable.

#### Thalamic relay (RE) neuron model

Model equations and parameters are taken from [4]

- **Classification of gating variables as fast (** 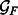 **), slow (** 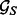 **), and adaptation (** 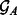 **) variables.** The model includes fast sodium channels 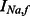, delayed-rectifier potassium channels 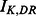 and T-type calcium channels 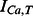. We classify model variables as follows

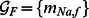

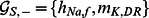
 and 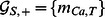

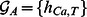

- **Balance equation and choice of the bifurcation parameter.** The only source of regenerative excitability is provided by T-type calcium channels. The associated balance equation reads

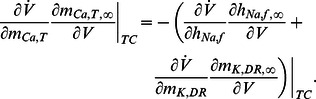



T-type calcium channels are dynamically regulated by a (slow) voltage-gated inactivation 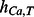. We use this variable as the bifurcation parameter (*i.e.* 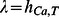).

**Figure 9:**
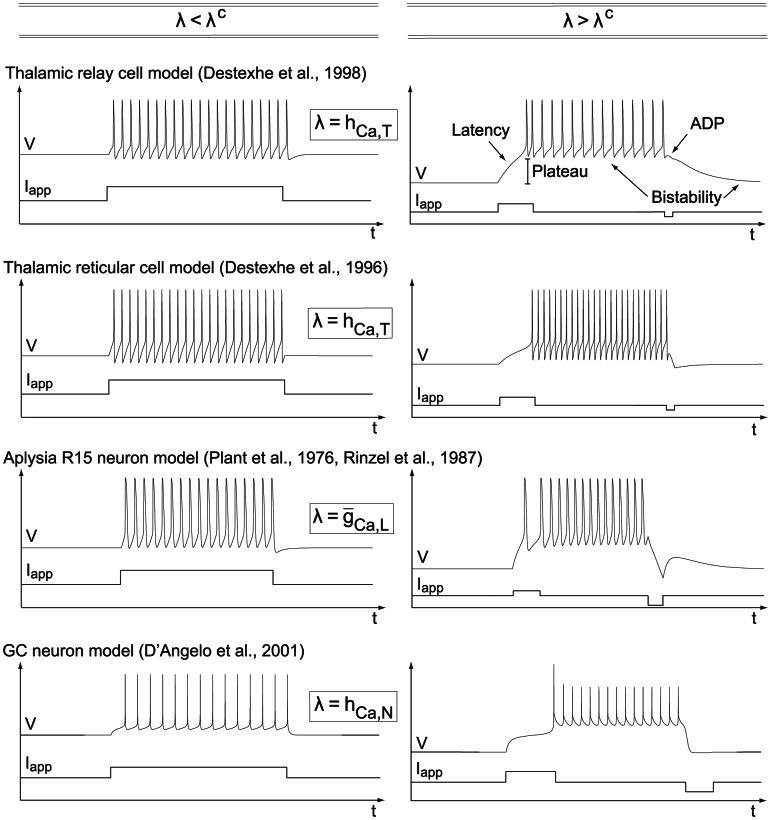
The same mathematical bifurcation in different conductance-base models causes the same switch in electrophysiological signatures. The figure shows the electrophysiological responses of various conductance-based models to step inputs of excitatory/inhibitory current when the bifurcation parameter 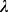 is lower (left) or higher (right) than the critical value 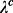. This bifurcation parameter can be either the density or the inactivation variable of a regenerative channel. Other adaptation variables are set to constant values chosen in physiological ranges (see text). For 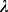 lower (resp. higher) than the critical value 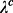, all models exhibit electrophysiological signatures of restorative (resp. regenerative) excitability. Numerical values of the parameter 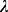 in the different plots are as follows. Thalamic relay cell: left 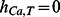, right 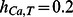. Thalamic reticular cell: left 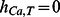, right 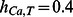. Aplysia R15 neuron: left 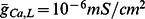, right 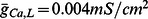. GC neuron: left 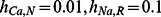, right 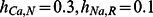.

Solving Steps (iii) and (iv) of the algorithm above gives the following results:
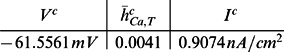
Since 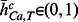, the model dynamically switches between restorative and regenerative excitability when 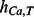 crosses the critical value, and the electrophysiological signatures are consistent with the excitability switches (see Fig. 9).

#### Thalamic reticular (RT) neuron model

Model equations and parameters are taken from [5], maximal conductances are adapted as in [11].

- **Classification of gating variables as fast (** 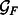 **), slow (** 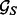 **), and adaptation (** 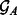 **) variables** The model includes fast sodium channels 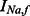, delayed-rectifier potassium channels 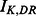 and T-type calcium channels 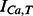. We classify model variables as follows

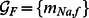

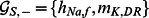
 and 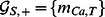

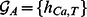

- **Balance equation and choice of the bifurcation parameter** The only source of regenerative excitability is provided T-type calcium channels. The associated balance equation has the same structure as for the thalamic relay neuron model considered above. Along the same line, we choose the T-type calcium channel inactivation 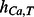 as the bifurcation parameter (*i.e.* 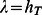).

Solving Steps (iii) and (iv) of the algorithm above gives the following results:
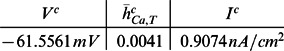



As in the case of the thalamic relay neuron model, the T-type calcium channel inactivation generates a dynamical switch between restorative and regenerative excitability, significantly affecting neuron response to external inputs (Fig. 9).

#### Aplysia R15 neuron model

Model equations and parameters are taken from [6].

- **Classification of gating variables as fast (** 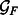 **), slow (** 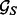 **), and adaptation (** 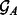 **) variables.** The model includes fast sodium channels 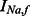, delayed-rectifier potassium channels 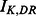, slow L-type calcium channels 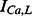 and calcium-activated potassium channels 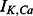. We classify model variables as follows

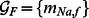

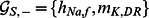
 and 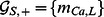

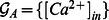

- The intracellular calcium conductance is fixed at 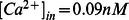.
- **Balance equation and choice of the bifurcation parameter.** As in the case of DA neurons, the source of regenerative excitability is provided by L-type calcium channels, and we take their maximal conductance 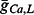 as the bifurcation parameter. The associated balance equation has the same structure as for the DA neuron model considered above.

Solving Steps (iii) and (iv) of the algorithm above gives the following results:
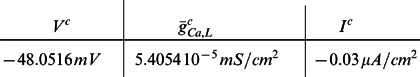
Comparing the critical value 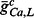 with the original value 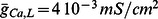 shows that the bursting model proposed in [6] exhibits strong regenerative excitability. Switches of electrophysiological signatures are illustrated in Fig. 9.

#### Cerebellar granule cell (GC) model

Model equations and parameters are taken from [7].

**Figure 10:**
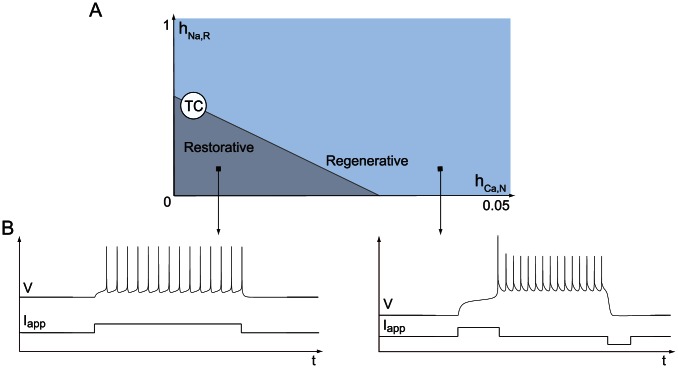
Joint variations of the inactivation gates of 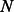-type calcium channels and resurgent sodium channels induce excitability switches in cerebellar granule cells. **A.** Two parameter bifurcation diagram of the mode with 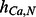 and 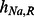 as bifurcation parameters. TC denotes a branch of transcritical bifurcations detected following the algorithm in Table 2. **B.** Electrophysiological responses of the model to step inputs of excitatory/inhibitory current: left 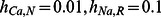, right 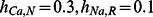.

- **Classification of gating variables as fast (** 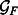 **), slow (** 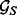 **), and adaptation (** 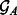 **) variables.** The model includes the following ion channels: fast (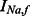), persistent (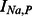) and resurgent sodium channels (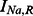) ; N-type calcium channels (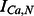) ; delayed rectifier (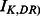, A-type (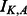), inward rectifier (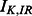), calcium activated (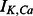) and slow potassium channels (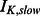). We classify model variables as follows

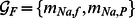

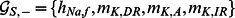
 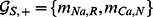

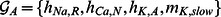
 and
- We set the persistent and calcium-activated currents to zero (these two channels do not impact excitability type as anticipated by our classification and shown by D'Angelo and colleagues [7]). The inactivation of the A-type potassium current is fixed at 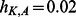 and the activation of the slow potassium current is fixed at 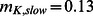.
- **Balance equation and choice of the bifurcation parameter.** The neuron model possesses two sources of regenerative excitability: resurgent sodium channels and N-type calcium channels. As in the case of T-type calcium channels mentioned above, these two channels possess an inactivation gate, which is used as the bifurcation parameter. We apply our algorithm by varying the parameter of one regenerative current while fixing the other at different values. This permits to draw an approximated hypersurface in the 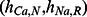 plane at which the balance equation is satisfied and the model undergoes the transcritical bifurcation and the associated excitability switch. The two balance equations read, respectively:
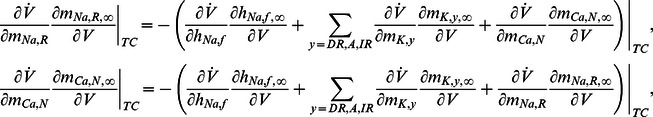
Solving Steps (iii) and (iv) of the algorithm, one obtains the parameter chart in Fig. 10. These results show that both channels can induce a dynamical switch in excitability. However, the N-type calcium channel contributes much more to regenerative excitability than the resurgent sodium channel in this model: as soon as 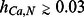 the model is in regenerative excitability for all values of 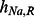. On the contrary, when 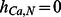 the inactivation of resurgent sodium channel should be reduced at least by a factor two for the model to exhibit regenerative excitability.

### The balance equation determines a switch from restorative to regenerative excitability

As illustrated in Figure 4, the significance of the transcritical bifurcation is that it delineates in the parameter space the boundary of a specific type of excitability and that this boundary is determined by a simple physiological balance (Eq. 9) between restorative and regenerative channels.

Specific to regenerative excitability is the bistable phase portrait of Fig. 1, right. For the six analyzed conductance-based models, our bifurcation analysis of the full model confirms the existence of a bistable range beyond the transcritical bifurcation, where a regenerative resting state and a spiking limit cycle coexist. In each case, the bistability range is obtained for the nominal time scales of the published model and is robust to a variation of time scales. In each case, the bistabilty range is also neuromodulated, that is, determined by conductance parameters that are known to vary in slower time scales and/or across neurons of a same type.

**Figure 11:**
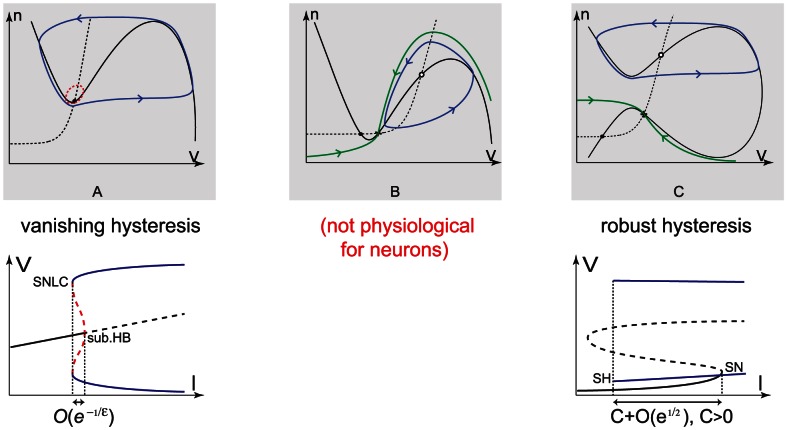
Three bistable phase portraits of model (1) and cartoon of the associated hysteretic bifurcation diagrams. In the phase portraits, a solid curve denotes the 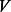-nullcline, whereas a dashed curve denotes the 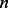-nullcline. Stable fixed points are depicted as filled circles, whereas unstable as circles and saddle points as cross. Stable limit cycles are drawn as solid oriented blue curves, whereas unstable as red dashed curves. The stable manifolds of saddle points are depicted as green oriented curves. In bifurcation diagrams, a solid curve denotes branches of stable fixed points, whereas a dashed curve denotes branches of unstable or saddle points. Branches of stable limit cycles are depicted as blue curves, whereas branches of unstable limit cycles as red dashed curves. *sub.HB* denotes a subcritical Hopf bifurcation, *SNLC* a saddle-node limit cycles bifurcation, *SN* a saddle-node bifurcation, and *SH* a saddle-homoclinic bifurcation. **A–B.** Restorative bistability. **A.** Subcritical Hopf bifurcation. Hysteresis vanishes exponentially fast as timescale separation increases. **B.** Restorative saddle-homoclinic bifurcation. Not physiological because it violates the time scale separation between 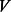 and 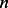. **C.** Regenerative bistability ruled by a regenerative saddle-homoclinic bifurcation. Hysteresis is barely affected by time-scale separation.

It is important to distinguish this robust and physiologically regulated bistability from other types of bistability that can be encountered in conductance-based models. Figure 11 qualitatively illustrates three typical bistable phase portraits associated to the planar model (1) that exhibit the coexistence of a stable resting state and of a spiking limit cycle. The first two are associated to restorative excitability and are extensively studied in the literature. See, e.g. [13], [14], and references therein. Only the third one is associated to regenerative excitability.

The three bistable phase portraits share the common feature of “hard excitation”: as the amplitude of a step input depolarizing current is increased, the response of the neuron abruptly switches from no oscillation to high frequency spiking. Following the historical classification of Hodgkin [15], the three situations correspond to Class II neurons, as opposed to Class I neurons for which the spiking frequency gradually increases with the depolarizing current amplitude.

Hard excitation can be a manifestation either of a switch-like monostable bifurcation diagram or of a hysteretic bistable bifurcation diagram. By definition, the three bistable phase portraits in Figure 11 give rise to hysteretic bifurcation diagrams. But for the two bistable phase portraits associated to restorative excitability, the hysteresis is highly dependent on the time scale separation, *i.e.*, the ratio 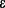 between the fast and slow time constants. In the case of the first phase portrait (subcritical Hopf bifurcation), asymptotic analysis shows that the hysteresis vanishes as 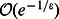. In the case of second phase portrait (saddle-homoclinic bifurcation), the situation is even worse because for small 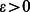 the system necessarily undergoes a monostable saddle-node on invariant circle bifurcation. In fact, the second phase portrait is not physiological for neuron conductance based models. For instance, in the hypothetical 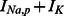 conductance-based model considered in [14, Fig. 6.44], the time constant of the potassium activation must be set below 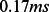 to create a saddle-homoclinic bifurcation, which is roughly 40 times smaller than its physiological value and even smaller than the fast time constant. A geometric proof of the generality of this fact is provided in [1]. The conclusion is that hysteresis associated to restorative excitability is at best very small (if any) in physiologically plausible conductance based models, which makes their electrophysiological signatures similar to those associated to a switch-like monostable bifurcation diagram.

In sharp contrast, the hysteresis associated to regenerative excitability is barely affected by the time-scale separation. Instead it is regulated by conductance parameters whose modulation is physiological (for instance, a regenerative ion channel density). The extended hysteresis is what determines the specific electrophysiological signature of regenerative excitability: a pronounced spike latency, a possible plateau oscillation, and an after depolarization potential. As a consequence, those features cannot be robustly reproduced in physiologically plausible conductance based models of restorative excitability. Because those features are important markers of modern electrophysiology [22], [23], the distinction between restorative and regenerative excitability seems physiologically relevant, beyond the possible shared feature of hard excitation.

In conclusion, the bistability associated to regenerative excitability is specific in that it produces a robust electrophysiological signature in physiologically plausible parameter ranges and consistent with many experimental observations. It is in that sense that the balance equation delineates a switch of excitability of physiological relevance.

### A refined classification of neuronal excitability

Early in the history of neurodynamics [15], Hodgkin proposed a classification of excitability in three different classes:

*Class I*: The spiking frequency vs. input current amplitude (f/I) curve is continuous, *i.e.*, the spiking frequency continuously increases from zero to high-frequency firing as the input current amplitude rises. Class I excitability is also referred to as “soft” excitation.

*Class II*: The f/I is discontinuous, *i.e.*, the spiking frequency abruptly switches from zero to high-frequency firing as the amplitude of the applied current is raised above a certain threshold. Class II excitability is also referred to as “hard” excitation.

*Class III*: The spiking frequency is zero for all amplitudes of the applied current. Transient action potentials can be generated in response to high-frequency stimuli.

Because regenerative excitability exhibits hard excitation, it is a physiologically distinct subtype of Class II excitability.

**Figure 12:**
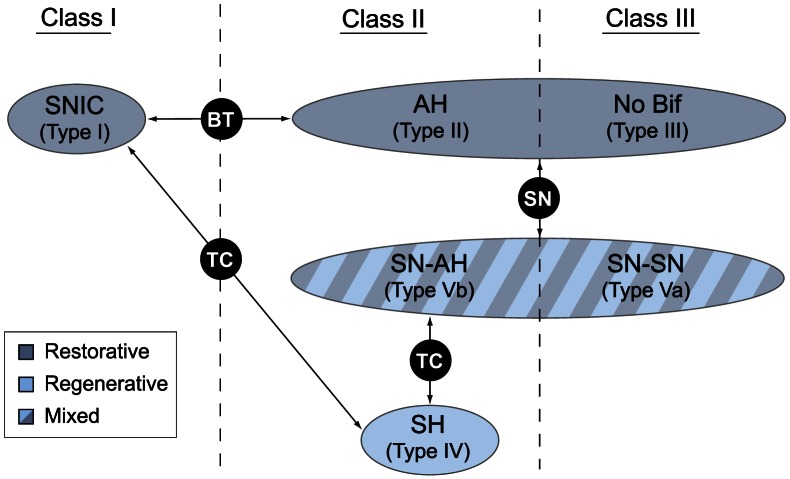
The various bifurcations associated to different types of neuronal excitability. *SNIC*: saddle-node on invariant circle; *BT*: Bogdanov-Takens; *AH*: Andronov-Hopf; *SN*: saddle-node; *TC*: transcritical; *SH*: saddle-homoclinic. See also [1] for more detailed definitions and properties of excitability Types I-V and associated transition bifurcations in a planar neuron model. Class I excitability occurs in the neighborhood of a SNIC bifurcation [12] and is purely restorative. Class II excitability can be either restorative in which case the stable equilibrium looses stability in a subcritical Hopf bifurcation (Type II in [12]) or regenerative in which case a stable equilibrium coexists with a stable limit cycle over a robust bistable range organized by a (singularly perturbed) saddle homoclinic bifurcation (Type IV in [1]). In a small parameter range, class II excitability can also exhibit a mixed type (Type Vb in [1]), where a regenerative "down" stable equilibrium coexists with a "up" restorative stable equilibrium or limit cycle. Stability of those attractors is lost either in saddle-node or Hopf bifurcations. Class III excitability can be either restorative (a monostable equilibrium) or exhibits a mixed type (Type Va in [1]), where a regenerative down stable equilibrium coexists with a restorative up stable equilibrium. Both attractors loose stability in a saddle-node bifurcation. The transition to regenerative excitability is always through a transcritical bifurcation.

Bifurcation theory helps relating this physiological classification to mathematical signatures of the associated neuron models. Distinct bifurcations delineate the different excitability classes as well as the different excitability mechanisms within a given class. They are summarized in Fig. 12.

Ermentrout [12] showed that Class I excitability arises from a saddle-node on invariant circle bifurcation, whereas Class II excitability arises from a Hopf bifurcation. Both bifurcations correspond to examples of restorative excitability in the terminology of the present paper and the transition between Class I and II is governed by a Bogdanov-Takens bifurcation.

Our recent paper [1] further expands this classification to account for regenerative excitability. Regenerative excitability (called Type IV in [1]) arises from a (singularly perturbed) saddle-homoclinic bifurcation and the transition from restorative to regenerative excitability always involves a transcritical bifurcation.

## Discussion

### A simple and robust balance equation identifies a transcritical bifurcation in arbitrary conductance based models

Motivated by a geometric analysis of a qualitative phase portrait, we have proposed an algorithm that easily detects a transcritical bifurcation in arbitrary conductance based models. Owing to the special structure of such models, the algorithm leads to solving an algebraic equation of remarkable simplicity and physiological relevance: a balance between slow restorative and slow regenerative ion channels. The condition is also robust because the balance is independent of the detailed kinetics, even though it critically relies on a classification of variables in three well separated time-scales, in good agreement with what is known on ion channels kinetics [8].

### The ubiquity of a transcritical bifurcation in conductance-based models

The detection of the transcritical bifurcation relies on the sole existence of a physiological balance between restorative and regenerative ion channels. Given that all neuronal models possess restorative sodium and potassium channels, this implies that a transcritical bifurcation exists in every conductance-based model that possesses at least one regenerative ion channel. Moreover, the channel balance, and therefore the TC bifurcation, are readily detectable in a model of arbitrary dimension (both in the state and parameters): the balance (9) simply defines a hypersurface in the parameter space that can algebraically be tracked under arbitrary parameter variations. An illustration was provided on the GC model above.

In spite of its ubiquity and of its physiological significance, we are not aware of an earlier reference to a transcritical bifurcation in conductance based models. A reason for this omission might be accidental: there are no regenerative channels in the seminal model of Hodgkin and Huxley (unless one modifies the potassium resting potential 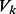) and this model has been the inspiration of most mathematical analyses of conductance-based models.

For the same reason, it seems physiologically relevant to distinguish between restorative and regenerative excitability beyond Hodgkin's classification of Class I (“soft”) and Class II (“hard”) excitability. Regenerative (and restorative) excitability faithfully capture the presence (or the absence) of specific electrophysiological signatures of modern electrophysiology such as spike latency, afterdepolarization potentials, or robust coexistence of resting state and repetitive spikes.

### A single mathematical prediction applies to many distinct physiological observations

Although purely mathematical in nature, the transcritical bifurcation has a remarkable predictive value in several published conductance based models. In each of the six analysed models, the proposed algorithm identifies a physiological parameter that acts as a tuner of neuronal excitability in a physiologically plausible range and in full agreement with existing experimental data. At the same time, the distinct nature of the regulating parameter, which can be either the maximal conductance or the inactivation gating variable of a regenerative ion channel depending on the neuron model, is associated to distinctly different regulation mechanisms.

### Reduced modeling should retain the balancing channel

The classification of gating variables in three distinct time scales is an essential modeling step both for the proposed algorithm and for the reduction of full conductance-based models to low-dimensional models that can be used in population studies [24]. Despite the inherent robustness of time-scale separation analysis, this classification is a limitation of the proposed approach if the model contains ion channels with poorly known kinetics. When all slow ion channels are properly identified, they can be aggregated in a single slow variable to lead to a second order model of the type (1), where the single parameter 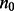 captures the restorative or regenerative nature of the aggregated slow variable. Further reduction to a one-dimensional hybrid model with reset is possible thanks to the time-scale separation between the voltage 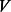 and the slow variable 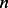. This reduction is illustrated in [11] on the thalamic TC neuron and the reduced model remarkably retains the switch of excitability of its high-dimensional counterpart. In contrast, a reduced model will lose the switch of excitability of the full conductance-based model when a regenerative ion channel is treated as a fast gating variable. It is for instance common in model reduction to set the activation of a calcium channel to steady state. This amounts to treat the calcium activation as a fast variable, which makes the channel either “slow restorative” or “neutral” in the terminology of this paper. If the calcium channel is the only source of regenerative excitability, then the reduced model will not retain features of regenerative excitability.

### Neuronal excitability is regulated

In each of the analysed conductance-based models, the balance equation responsible for the switch of excitability is satisfied for a set of parameters that is close to the published parameter values. This observation supports the hypothesis that neuronal excitability is tightly regulated by molecular mechanisms and that the influence of the channel balance condition on neuronal excitability might play a role in neuronal signaling.

## Materials and Methods

### Numerical analysis

Numerical temporal traces of the different neurons (Figs. 1, 6, 8, 9) were reproduced by implementing in MATLAB (available at http://www.mathworks.com) the original models as described in the associated papers. The phase portraits in Figures 1 and 3 were hand-drawn using the Open Source vector graphics editor Inkscape (http://inkscape.org). The phase portraits in Figure 6 were numerically drawn with MATLAB by implementing the planar model in [10] and subsequently modified with Inkscape. The bifurcation diagrams in Figures 6, 7, and 8 were drawn by implementing the algorithm of Table 2 in MATLAB. No figure or part of figure was reproduced from other published works.

## Supporting Information

Supplementary Material S1 Algorithmic detection of a transcritical bifurcaton in conductance-based models.
(PDF)
