# Supplementary material for: A Balance Equation Determines a Switch in Neuronal Excitability
Source: PLoS Comput Biol. 2013 May 23;9(5):e1003040. doi: 10.1371/journal.pcbi.1003040 (PMC3662658; doi:10.1371/journal.pcbi.1003040)
Supplement: Supplementary Material S1 — Algorithmic detection of a transcritical bifurcaton in conductance-based models. (PDF) [file pcbi.1003040.s001.pdf]

A Balance Equation Determines a Switch in Neuronal Excitability

Supplementary material S1

Construction of the transcritical bifurcation in generic conductance-based models

Suppose that, given a bifurcation parameter $\mu \in \{\bar{g}_i, E_i\}_{i\in\mathcal{I}}$, there exists a solution $(V,\mu) = (V^c,\mu^c)$ satisfying the two degeneracy conditions (7) and (8). Then we claim that, posing

$$I^c = \sum_i \bar{g}_i m_{i\infty}^{a_i}(V^c) h_{i\infty}^{b_i}(V^c)(V^c - E_i),$$

which solves the fixed point equation, the model is at a transcritical bifurcation. The existence of the couple (V^c,μ^c) in the physiological range is shown for specific examples in the main document.

The center space E_c associated to the degeneracy conditions (7) and (8) is spanned by the vector

$$v_c = (1, \mathbf{k}), \quad \mathbf{k} := [k_x]_x, \quad k_x := \left.\frac{\partial x_\infty}{\partial V}\right|_{V=V^c},$$

where x runs all fast and slow gating variables in the model, that is $\mathbf{k}$ is the vector whose elements are the slopes of the (in)activation functions of all the fast and slow gating variables calculated at $V = V^c$. The associated center manifold $\mathcal{M}_c$ is exponentially attractive. Indeed, it can easily been shown that, when (7) and (8) are satisfied, the remaining nonzero eigenvalues of the Jacobian of (10) are all negative. To the first order, the dynamics on $\mathcal{M}_c$ is given by

$$C_m \dot{V}_c = -\sum_{i\in\mathcal{I}} \bar{g}_i \big(m_{i,\infty}(V^c) + k_{m_i}(V_c - V^c)\big)^{a_i} \big(h_{i,\infty}(V^c) + k_{h_i}(V_c - V^c)\big)^{b_i} (V_c - E_i) + I^c + I_{app}$$

Consider the affine reparametrization

$$\tilde{\mu} = \mu, \quad \tilde{I}_{app} = I_{app} + \left.\frac{\partial \dot{V}_c}{\partial \mu}\right|_{\substack{\mu=\mu^c\\ V=V^c}} (\mu - \mu^c). \tag{S1}$$

It is easy to show that, in the affine reparametrization (S1), the center manifold dynamics satisfy

$$\left.\dot{V}_c\right|_{\substack{\mu=\mu^c\\ V=V^c}} = \left.\frac{\partial \dot{V}_c}{\partial V}\right|_{\substack{\mu=\mu^c\\ V=V^c}} = \left.\frac{\partial \dot{V}_c}{\partial \tilde{\mu}}\right|_{\substack{\mu=\mu^c\\ V=V^c}} = 0$$

corresponding to the defining condition of a transcritical bifurcation (see [1, Page 367]).

Supplementary references

[1] Seydel R (2010) Practical bifurcation and stability analysis, volume 5 of *Interdisciplinary Applied Mathematics*. New York: Springer-Verlag, third edition.
